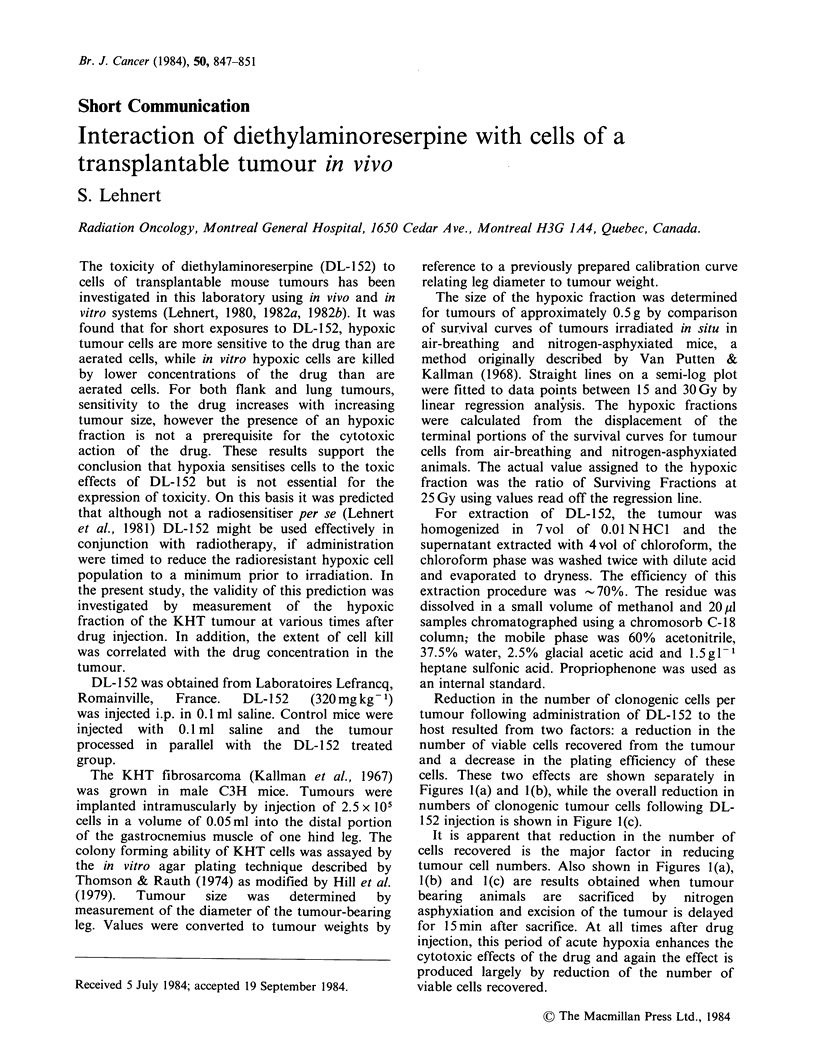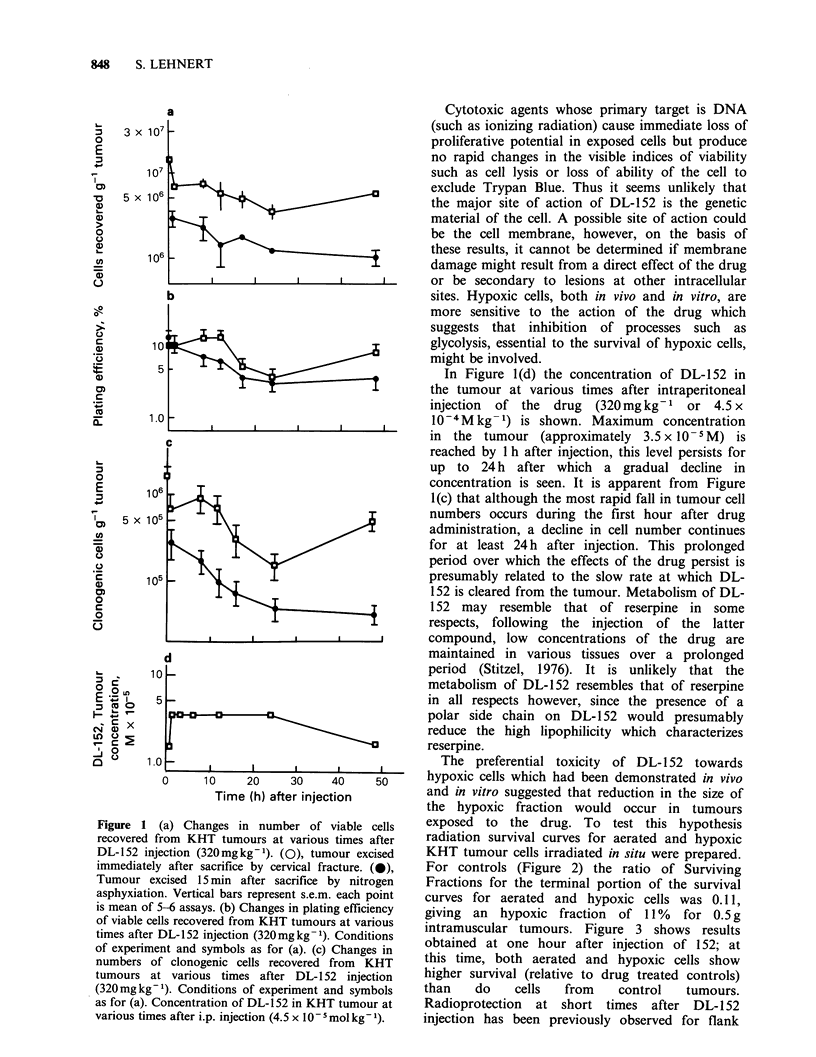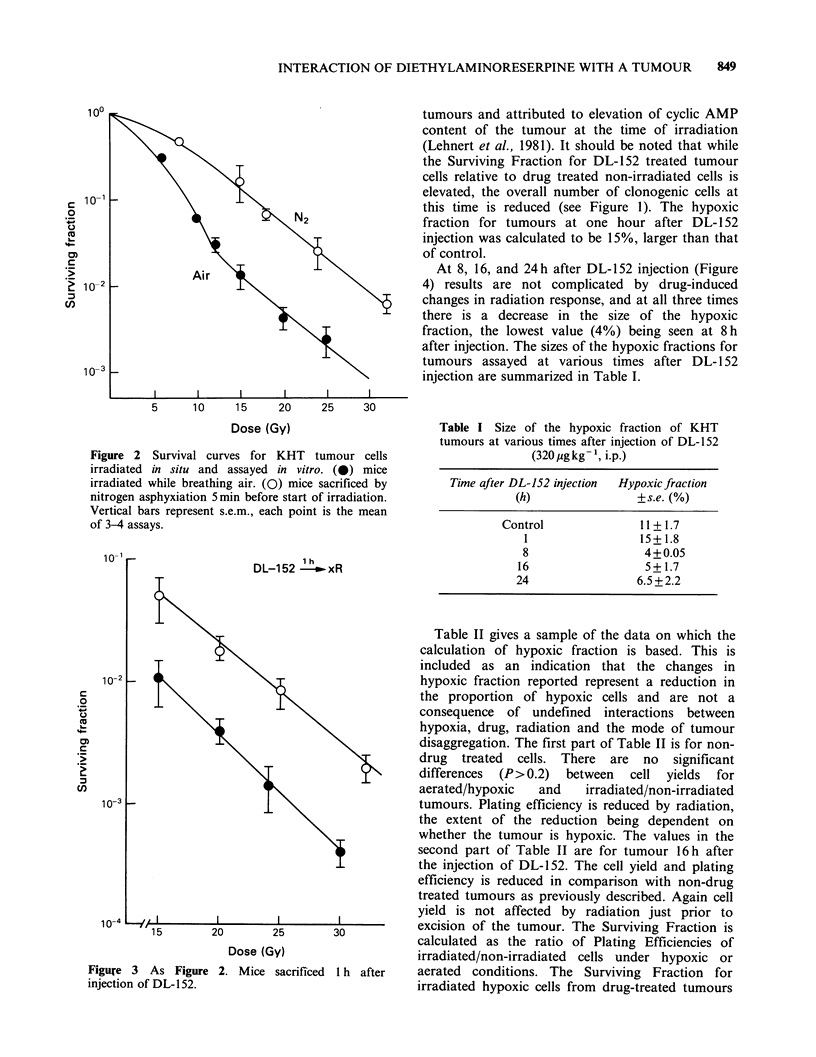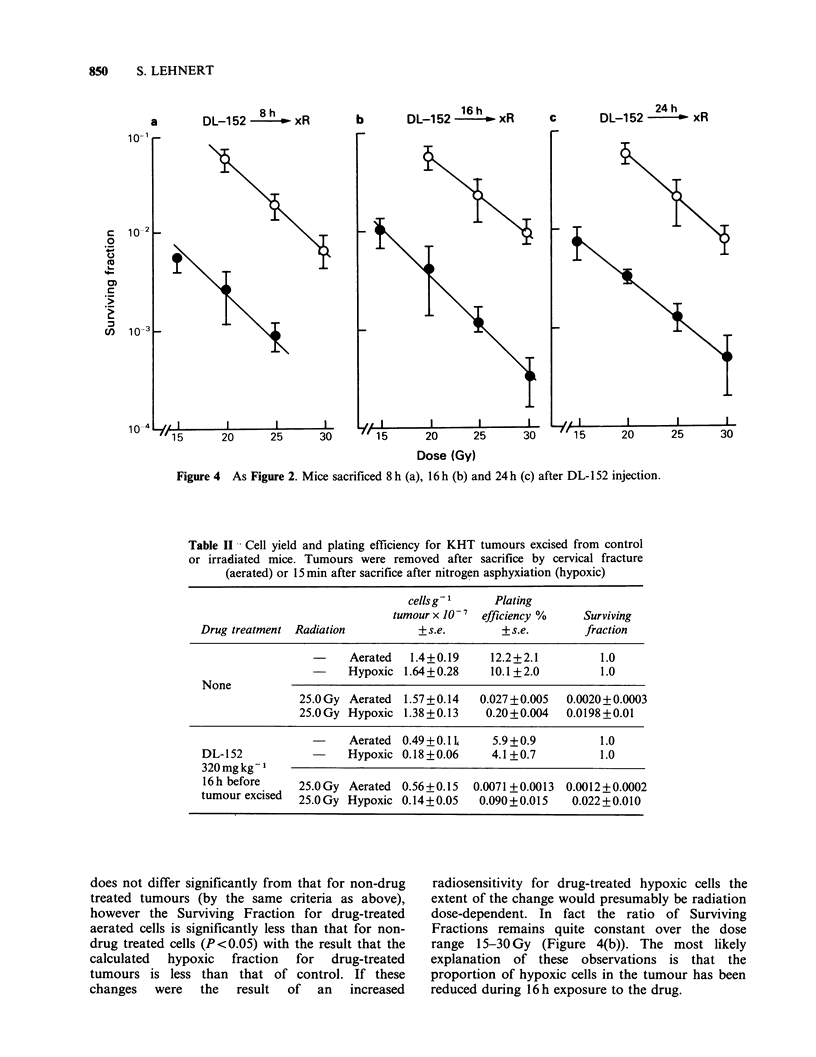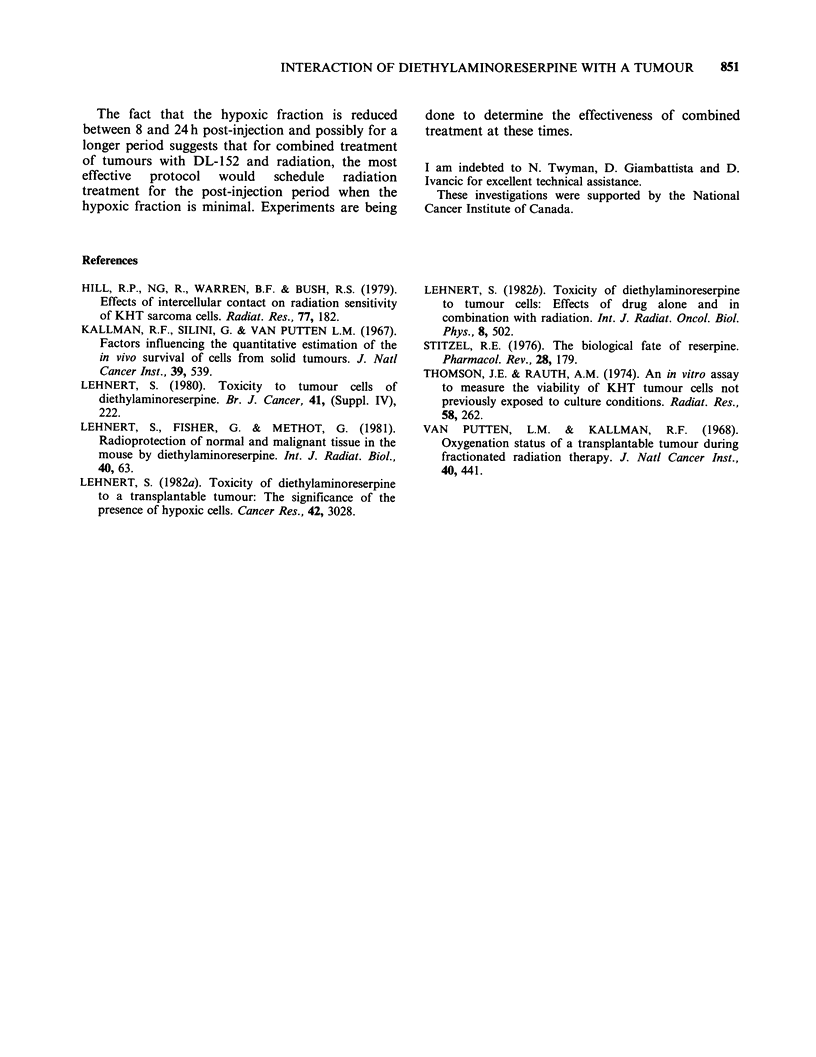# Interaction of diethylaminoreserpine with cells of a transplantable tumour in vivo.

**DOI:** 10.1038/bjc.1984.267

**Published:** 1984-12

**Authors:** S. Lehnert


*Br. J. Cancer* (1984), **50**, 847–851

### Short Communication

### Interaction of diethylaminoreserpine with cells of a transplantable tumour *in vivo*

S. Lehnert

*Radiation Oncology, Montreal General Hospital, 1650 Cedar Ave., Montreal H3G 1A4, Quebec, Canada.*

The toxicity of diethylaminoreserpine (DL-152) to cells of transplantable mouse tumours has been investigated in this laboratory using *in vivo* and *in vitro* systems (Lehnert, 1980, 1982*a*, 1982*b*). It was found that for short exposures to DL-152, hypoxic tumour cells are more sensitive to the drug than are aerated cells, while *in vitro* hypoxic cells are killed by lower concentrations of the drug than are aerated cells. For both flank and lung tumours, sensitivity to the drug increases with increasing tumour size, however the presence of an hypoxic fraction is not a prerequisite for the cytotoxic action of the drug. These results support the conclusion that hypoxia sensitises cells to the toxic effects of DL-152 but is not essential for the expression of toxicity. On this basis it was predicted that although not a radiosensitiser *per se* (Lehnert *et al.*, 1981) DL-152 might be used effectively in conjunction with radiotherapy, if administration were timed to reduce the radioresistant hypoxic cell population to a minimum prior to irradiation. In the present study, the validity of this prediction was investigated by measurement of the hypoxic fraction of the KHT tumour at various times after drug injection. In addition, the extent of cell kill was correlated with the drug concentration in the tumour.

DL-152 was obtained from Laboratoires Lefrancq, Romainville, France. DL-152 ($320\,mg\,kg^{-1}$) was injected i.p. in 0.1 ml saline. Control mice were injected with 0.1 ml saline and the tumour processed in parallel with the DL-152 treated group.

The KHT fibrosarcoma (Kallman *et al.*, 1967) was grown in male C3H mice. Tumours were implanted intramuscularly by injection of $2.5\times10^{5}$ cells in a volume of 0.05 ml into the distal portion of the gastrocnemius muscle of one hind leg. The colony forming ability of KHT cells was assayed by the *in vitro* agar plating technique described by Thomson & Rauth (1974) as modified by Hill *et al.* (1979). Tumour size was determined by measurement of the diameter of the tumour-bearing leg. Values were converted to tumour weights by reference to a previously prepared calibration curve relating leg diameter to tumour weight.

The size of the hypoxic fraction was determined for tumours of approximately 0.5 g by comparison of survival curves of tumours irradiated *in situ* in air-breathing and nitrogen-asphyxiated mice, a method originally described by Van Putten & Kallman (1968). Straight lines on a semi-log plot were fitted to data points between 15 and 30 Gy by linear regression analysis. The hypoxic fractions were calculated from the displacement of the terminal portions of the survival curves for tumour cells from air-breathing and nitrogen-asphyxiated animals. The actual value assigned to the hypoxic fraction was the ratio of Surviving Fractions at 25 Gy using values read off the regression line.

For extraction of DL-152, the tumour was homogenized in 7 vol of 0.01 N HCl and the supernatant extracted with 4 vol of chloroform, the chloroform phase was washed twice with dilute acid and evaporated to dryness. The efficiency of this extraction procedure was ~70%. The residue was dissolved in a small volume of methanol and 20 $\mu$l samples chromatographed using a chromosorb C-18 column; the mobile phase was 60% acetonitrile, 37.5% water, 2.5% glacial acetic acid and $1.5\,g\,l^{-1}$ heptane sulfonic acid. Propriophenone was used as an internal standard.

Reduction in the number of clonogenic cells per tumour following administration of DL-152 to the host resulted from two factors: a reduction in the number of viable cells recovered from the tumour and a decrease in the plating efficiency of these cells. These two effects are shown separately in Figures 1(a) and 1(b), while the overall reduction in numbers of clonogenic tumour cells following DL-152 injection is shown in Figure 1(c).

It is apparent that reduction in the number of cells recovered is the major factor in reducing tumour cell numbers. Also shown in Figures 1(a), 1(b) and 1(c) are results obtained when tumour bearing animals are sacrificed by nitrogen asphyxiation and excision of the tumour is delayed for 15 min after sacrifice. At all times after drug injection, this period of acute hypoxia enhances the cytotoxic effects of the drug and again the effect is produced largely by reduction of the number of viable cells recovered.

Received 5 July 1984; accepted 19 September 1984.

© The Macmillan Press Ltd., 1984

**Figure 1** (a) Changes in number of viable cells recovered from KHT tumours at various times after DL-152 injection (320 mg kg$^{-1}$). (○), tumour excised immediately after sacrifice by cervical fracture. (●), Tumour excised 15 min after sacrifice by nitrogen asphyxiation. Vertical bars represent s.e.m. each point is mean of 5–6 assays. (b) Changes in plating efficiency of viable cells recovered from KHT tumours at various times after DL-152 injection (320 mg kg$^{-1}$). Conditions of experiment and symbols as for (a). (c) Changes in numbers of clonogenic cells recovered from KHT tumours at various times after DL-152 injection (320 mg kg$^{-1}$). Conditions of experiment and symbols as for (a). Concentration of DL-152 in KHT tumour at various times after i.p. injection ($4.5 \times 10^{-5}$ mol kg$^{-1}$).

Cytotoxic agents whose primary target is DNA (such as ionizing radiation) cause immediate loss of proliferative potential in exposed cells but produce no rapid changes in the visible indices of viability such as cell lysis or loss of ability of the cell to exclude Trypan Blue. Thus it seems unlikely that the major site of action of DL-152 is the genetic material of the cell. A possible site of action could be the cell membrane, however, on the basis of these results, it cannot be determined if membrane damage might result from a direct effect of the drug or be secondary to lesions at other intracellular sites. Hypoxic cells, both *in vivo* and *in vitro*, are more sensitive to the action of the drug which suggests that inhibition of processes such as glycolysis, essential to the survival of hypoxic cells, might be involved.

In Figure 1(d) the concentration of DL-152 in the tumour at various times after intraperitoneal injection of the drug (320 mg kg$^{-1}$ or $4.5 \times 10^{-4}$ M kg$^{-1}$) is shown. Maximum concentration in the tumour (approximately $3.5 \times 10^{-5}$ M) is reached by 1 h after injection, this level persists for up to 24 h after which a gradual decline in concentration is seen. It is apparent from Figure 1(c) that although the most rapid fall in tumour cell numbers occurs during the first hour after drug administration, a decline in cell number continues for at least 24 h after injection. This prolonged period over which the effects of the drug persist is presumably related to the slow rate at which DL-152 is cleared from the tumour. Metabolism of DL-152 may resemble that of reserpine in some respects, following the injection of the latter compound, low concentrations of the drug are maintained in various tissues over a prolonged period (Stitzel, 1976). It is unlikely that the metabolism of DL-152 resembles that of reserpine in all respects however, since the presence of a polar side chain on DL-152 would presumably reduce the high lipophilicity which characterizes reserpine.

The preferential toxicity of DL-152 towards hypoxic cells which had been demonstrated *in vivo* and *in vitro* suggested that reduction in the size of the hypoxic fraction would occur in tumours exposed to the drug. To test this hypothesis radiation survival curves for aerated and hypoxic KHT tumour cells irradiated *in situ* were prepared. For controls (Figure 2) the ratio of Surviving Fractions for the terminal portion of the survival curves for aerated and hypoxic cells was 0.11, giving an hypoxic fraction of 11% for 0.5 g intramuscular tumours. Figure 3 shows results obtained at one hour after injection of 152; at this time, both aerated and hypoxic cells show higher survival (relative to drug treated controls) than do cells from control tumours. Radioprotection at short times after DL-152 injection has been previously observed for flank

**Figure 2** Survival curves for KHT tumour cells irradiated *in situ* and assayed *in vitro*. (●) mice irradiated while breathing air. (○) mice sacrificed by nitrogen asphyxiation 5 min before start of irradiation. Vertical bars represent s.e.m., each point is the mean of 3–4 assays.

**Figure 3** As **Figure 2**. Mice sacrificed 1 h after injection of DL-152.

tumours and attributed to elevation of cyclic AMP content of the tumour at the time of irradiation (Lehnert *et al.*, 1981). It should be noted that while the Surviving Fraction for DL-152 treated tumour cells relative to drug treated non-irradiated cells is elevated, the overall number of clonogenic cells at this time is reduced (see Figure 1). The hypoxic fraction for tumours at one hour after DL-152 injection was calculated to be 15%, larger than that of control.

At 8, 16, and 24 h after DL-152 injection (Figure 4) results are not complicated by drug-induced changes in radiation response, and at all three times there is a decrease in the size of the hypoxic fraction, the lowest value (4%) being seen at 8 h after injection. The sizes of the hypoxic fractions for tumours assayed at various times after DL-152 injection are summarized in Table I.

**Table I** Size of the hypoxic fraction of KHT tumours at various times after injection of DL-152 (320 $\mu g\,kg^{-1}$, i.p.)

| *Time after DL-152 injection* (*h*) | *Hypoxic fraction* $\pm$*s.e.* (%) |
|---|---|
| Control | 11 ± 1.7 |
| 1 | 15 ± 1.8 |
| 8 | 4 ± 0.05 |
| 16 | 5 ± 1.7 |
| 24 | 6.5 ± 2.2 |

Table II gives a sample of the data on which the calculation of hypoxic fraction is based. This is included as an indication that the changes in hypoxic fraction reported represent a reduction in the proportion of hypoxic cells and are not a consequence of undefined interactions between hypoxia, drug, radiation and the mode of tumour disaggregation. The first part of Table II is for non-drug treated cells. There are no significant differences ($P > 0.2$) between cell yields for aerated/hypoxic and irradiated/non-irradiated tumours. Plating efficiency is reduced by radiation, the extent of the reduction being dependent on whether the tumour is hypoxic. The values in the second part of Table II are for tumour 16 h after the injection of DL-152. The cell yield and plating efficiency is reduced in comparison with non-drug treated tumours as previously described. Again cell yield is not affected by radiation just prior to excision of the tumour. The Surviving Fraction is calculated as the ratio of Plating Efficiencies of irradiated/non-irradiated cells under hypoxic or aerated conditions. The Surviving Fraction for irradiated hypoxic cells from drug-treated tumours

**Figure 4** As **Figure 2**. Mice sacrificed 8 h (a), 16 h (b) and 24 h (c) after DL-152 injection.

**Table II** Cell yield and plating efficiency for KHT tumours excised from control or irradiated mice. Tumours were removed after sacrifice by cervical fracture (aerated) or 15 min after sacrifice after nitrogen asphyxiation (hypoxic)

| *Drug treatment* | *Radiation* | | *cells g$^{-1}$ tumour × 10$^{-7}$ ±s.e.* | *Plating efficiency % ±s.e.* | *Surviving fraction* |
|---|---|---|---|---|---|
| None | — | Aerated | 1.4±0.19 | 12.2±2.1 | 1.0 |
| | — | Hypoxic | 1.64±0.28 | 10.1±2.0 | 1.0 |
| | 25.0 Gy | Aerated | 1.57±0.14 | 0.027±0.005 | 0.0020±0.0003 |
| | 25.0 Gy | Hypoxic | 1.38±0.13 | 0.20±0.004 | 0.0198±0.01 |
| DL-152 320 mg kg$^{-1}$ 16 h before tumour excised | — | Aerated | 0.49±0.11 | 5.9±0.9 | 1.0 |
| | — | Hypoxic | 0.18±0.06 | 4.1±0.7 | 1.0 |
| | 25.0 Gy | Aerated | 0.56±0.15 | 0.0071±0.0013 | 0.0012±0.0002 |
| | 25.0 Gy | Hypoxic | 0.14±0.05 | 0.090±0.015 | 0.022±0.010 |

does not differ significantly from that for non-drug treated tumours (by the same criteria as above), however the Surviving Fraction for drug-treated aerated cells is significantly less than that for non-drug treated cells ($P<0.05$) with the result that the calculated hypoxic fraction for drug-treated tumours is less than that of control. If these changes were the result of an increased radiosensitivity for drug-treated hypoxic cells the extent of the change would presumably be radiation dose-dependent. In fact the ratio of Surviving Fractions remains quite constant over the dose range 15–30 Gy (Figure 4(b)). The most likely explanation of these observations is that the proportion of hypoxic cells in the tumour has been reduced during 16 h exposure to the drug.

The fact that the hypoxic fraction is reduced between 8 and 24 h post-injection and possibly for a longer period suggests that for combined treatment of tumours with DL-152 and radiation, the most effective protocol would schedule radiation treatment for the post-injection period when the hypoxic fraction is minimal. Experiments are being done to determine the effectiveness of combined treatment at these times.

I am indebted to N. Twyman, D. Giambattista and D. Ivancic for excellent technical assistance.

These investigations were supported by the National Cancer Institute of Canada.

**References**

HILL, R.P., NG, R., WARREN, B.F. & BUSH, R.S. (1979). Effects of intercellular contact on radiation sensitivity of KHT sarcoma cells. *Radiat. Res.*, **77**, 182.

KALLMAN, R.F., SILINI, G. & VAN PUTTEN L.M. (1967). Factors influencing the quantitative estimation of the *in vivo* survival of cells from solid tumours. *J. Natl Cancer Inst.*, **39**, 539.

LEHNERT, S. (1980). Toxicity to tumour cells of diethylaminoreserpine. *Br. J. Cancer*, **41**, (Suppl. IV), 222.

LEHNERT, S., FISHER, G. & METHOT, G. (1981). Radioprotection of normal and malignant tissue in the mouse by diethylaminoreserpine. *Int. J. Radiat. Biol.*, **40**, 63.

LEHNERT, S. (1982*a*). Toxicity of diethylaminoreserpine to a transplantable tumour: The significance of the presence of hypoxic cells. *Cancer Res.*, **42**, 3028.

LEHNERT, S. (1982*b*). Toxicity of diethylaminoreserpine to tumour cells: Effects of drug alone and in combination with radiation. *Int. J. Radiat. Oncol. Biol. Phys.*, **8**, 502.

STITZEL, R.E. (1976). The biological fate of reserpine. *Pharmacol. Rev.*, **28**, 179.

THOMSON, J.E. & RAUTH, A.M. (1974). An *in vitro* assay to measure the viability of KHT tumour cells not previously exposed to culture conditions. *Radiat. Res.*, **58**, 262.

VAN PUTTEN, L.M. & KALLMAN, R.F. (1968). Oxygenation status of a transplantable tumour during fractionated radiation therapy. *J. Natl Cancer Inst.*, **40**, 441.